# Sex-based differences in ocular trauma: a systematic review with narrative synthesis

**DOI:** 10.3389/fpubh.2026.1814050

**Published:** 2026-07-01

**Authors:** Marah Eltiti, Omar Nusair, Mohammad Soleimani

**Affiliations:** 1 Department of Ophthalmology, An-Najah National University, Nablus, Palestine; 2 Department of Ophthalmology, UNC Kittner Eye Center, University of North Carolina at Chapel Hill, Chapel Hill, NC, United States; 3 Department of Ophthalmology, University of North Carolina at Chapel Hill, Chapel Hill, NC, United States

**Keywords:** epidemiology, eye injury, gender differences, intraocular foreign body, ocular trauma, open-globe injury, sex differences, visual outcomes

## Abstract

**Background:**

Ocular trauma is a major cause of preventable visual impairment and blindness worldwide. Although sex-based differences in ocular trauma have been reported, the evidence has not been comprehensively synthesized across mechanisms, injury patterns, severity, and outcomes.

**Methods:**

We conducted a systematic review with narrative synthesis of studies published between January 2000 and December 2025. A structured search was performed in PubMed/MEDLINE, and reference lists of eligible articles were manually screened for additional studies. Original human epidemiologic or clinical studies were eligible if they focused on ocular trauma and reported sex-disaggregated data or direct comparisons between males and females relevant to epidemiology, mechanism, injury pattern, severity, management, or outcome. Reviews, meta-analyses, editorials, letters, conference abstracts, case reports, and mechanistic or preclinical studies were excluded from the formal review. Owing to substantial heterogeneity, findings were synthesized narratively. Forty-seven primary human studies met the inclusion criteria.

**Results:**

Males consistently accounted for most ocular trauma cases and were more likely to sustain occupational, sports-related, penetrating, and open-globe injuries, including intraocular foreign bodies, with greater posterior segment involvement and poorer visual outcomes. Females were more commonly represented in domestic, fall-related, blunt, and violence-related injuries. Boys showed higher pediatric injury rates, whereas older women were more vulnerable to fall-related trauma.

**Conclusion:**

Ocular trauma shows clinically meaningful sex-based differences that may inform prevention, risk stratification, and patient management.

## Introduction

Ocular trauma is a major global health problem and a leading cause of preventable visual impairment and blindness. The World Health Organization estimates that 43 million people are blind worldwide, with a further 295 million living with moderate-to-severe visual impairment (1). Traumatic injuries make a substantial contribution to this burden, with more than 55 million eye injuries occurring annually and at least 19 million individuals experiencing uniocular blindness due to trauma; approximately 1.6 million become bilaterally blind (2). Beyond its clinical consequences, ocular trauma carries a considerable socioeconomic burden through loss of productivity, treatment costs, and long-term disability. This burden is particularly severe in low-resource settings, where 60–80% of children who become blind may die within one to 2 years (3).

The prevalence and incidence of ocular trauma vary widely across populations, reflecting differences in age, occupational exposure, environmental context, and both biological sex and socially mediated gender roles. Population-based studies indicate that ocular trauma is common in community settings in the United States, and that individuals with a prior eye injury are at increased risk of reinjury over time (4, 5). Lifetime cumulative prevalence among adults older than 40 years has been reported to reach 21.1% in Melbourne, while community-based U.S. data similarly demonstrate a substantial burden of prior ocular injury (5, 6). Hospital discharge-based studies from Maryland, Scotland, and Singapore have reported annual incidence rates of severe ocular trauma in the range of approximately 8–13 cases per 100,000 population, largely reflecting more severe injuries requiring hospitalization (7–9). Multicenter clinical data from U.S. eye centers further highlight the demographic and clinical diversity of ocular trauma presentations in specialist settings (10). Broader surveillance and population-based studies have reported higher overall incidence estimates, capturing both minor and severe injuries (4–6). Globally, ocular trauma accounts for a meaningful proportion of emergency visits and contributes substantially to monocular and binocular visual loss (1, 2). The economic burden is also considerable, with important direct and indirect costs reported across both high- and middle-income settings (2, 6, 11).

A consistent finding across epidemiologic studies is a marked sex disparity in ocular trauma. Across many studies and settings, males account for the majority of reported ocular injuries, often representing approximately 65–90% of cases, regardless of geographic region or socioeconomic setting, largely owing to greater occupational exposure and risk-taking behavior (11–18). Koo et al. (14) demonstrated that males sustain more severe open-globe injuries and experience poorer visual outcomes than females. Global Burden of Disease analyses further identify male sex and younger age as major predictors of trauma-related visual disability, particularly in injuries involving intraocular foreign bodies (19). In contrast, females are more frequently represented in domestic, fall-related, chemical, and violence-related injury patterns, including ocular trauma associated with intimate partner violence and home-based injury settings (20–25). These patterns underscore a complex interaction between biologic, social, and behavioral factors in shaping sex-related risk, mechanism, and outcome.

Although many studies have examined individual aspects of ocular trauma, no recent systematic review has comprehensively synthesized the global evidence on sex differences across the full spectrum of injury characteristics and outcomes. Existing studies are often limited by single-center design, variation in injury classification, and incomplete sex-disaggregated reporting, all of which complicate comparisons across populations. Despite efforts to standardize ocular trauma terminology and classification, heterogeneity in definitions and reporting remains a challenge in the literature (26). A clearer understanding of sex-based differences in ocular trauma is therefore essential to inform targeted prevention strategies, optimize clinical management, and guide public health interventions.

Accordingly, this systematic review synthesizes global evidence on sex differences in ocular trauma published between 2000 and 2025, including differences in mechanisms of injury, injury type and severity, anatomical involvement, and visual outcomes, while also considering biologic and hormonal mechanisms in contextual interpretation. By integrating evidence across diverse geographic and socioeconomic settings, this review aims to support the adoption of sex-informed approaches to ocular trauma prevention, assessment, and management.

## Methods

This study was conducted as a systematic review with narrative synthesis to summarize the available evidence on sex- and gender-related differences in ocular trauma. A structured literature search was performed in PubMed/MEDLINE to identify relevant studies published between January 2000 and December 2025. The search strategy combined terms related to ocular trauma, including ocular trauma, eye injury, open globe injury, closed globe injury, and intraocular foreign body, with sex- and gender-related terms including male, female, sex differences, and gender differences. Reference lists of relevant articles were also manually screened to identify additional eligible studies.

Studies were eligible for formal inclusion if they were original human epidemiologic or clinical studies published within the predefined time frame and reported sex-disaggregated data or direct comparisons between males and females relevant to ocular trauma epidemiology, mechanisms, injury patterns, severity, management, or outcomes. Review articles, systematic reviews, meta-analyses, editorials, letters, conference abstracts, case reports, mechanistic or preclinical studies, classification papers, contextual papers not primarily focused on ocular trauma or direct sex-based comparison, articles lacking relevant sex-disaggregated data, and reports with duplicate populations or redundant reporting were excluded from the formal review. Selected contextual, biologic, and mechanistic references were cited in the Introduction and Discussion to support interpretation, but were not included in the formal systematic review dataset.

**Figure 1:**
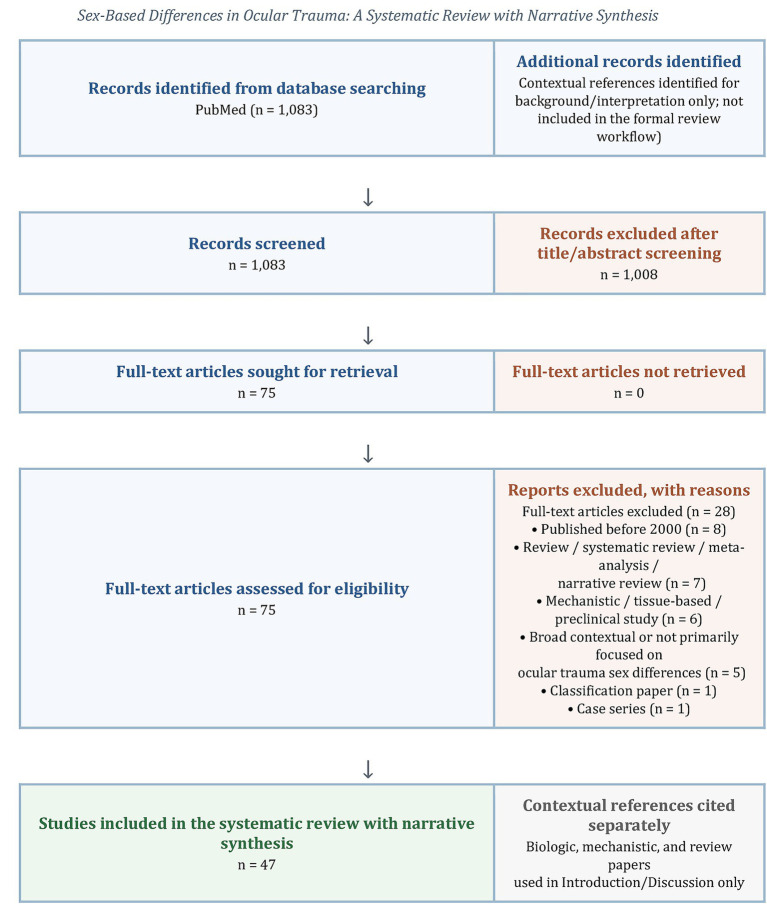
PRISMA 2020 flow diagram of the study selection process for the systematic review with narrative synthesis. The figure shows the identification, screening, eligibility assessment, and final inclusion of studies in the formal systematic review dataset, including records identified through PubMed/MEDLINE, full-text articles assessed for eligibility, excluded articles with reasons, and the final number of included studies.

After title and abstract screening, potentially eligible studies underwent full-text assessment. Because of substantial heterogeneity in study design, populations, injury classifications, and reported outcomes, the findings were synthesized narratively rather than quantitatively, and no meta-analysis was performed. The study-selection process is presented in Figure 1 using the PRISMA 2020 flow-diagram framework. A total of 1,083 records were identified through PubMed/MEDLINE. After title and abstract screening, 75 full-text articles were assessed for eligibility, and all were successfully retrieved. Of these, 28 full-text articles were excluded for the following reasons: publication before 2000 (*n* = 8), review/systematic review/meta-analysis/narrative review format (*n* = 7), mechanistic, tissue-based, or preclinical design (*n* = 6), lack of primary focus on ocular trauma sex differences or broad contextual scope (*n* = 5), classification paper (*n* = 1), and case-series design (*n* = 1). Ultimately, 47 original human studies met the predefined eligibility criteria and were included in the review.

The methodological quality of the included observational studies was assessed using the Newcastle-Ottawa Scale (NOS), adapted where appropriate for cross-sectional studies, and the results are presented in Table 1. A summary of the primary human studies included in the formal systematic review is provided in Table 2, while additional contextual, biologic, and mechanistic references cited for background and interpretation are summarized separately in Table 3. Ethical approval was not required because this review was based exclusively on previously published literature.

**Table 1:** Methodological quality assessment of included observational human studies using the Newcastle–Ottawa Scale (NOS).

Ref	Author, year	Study design	NOS score	Quality rating
(14)	Koo et al. (2005)	Retrospective cohort	8/9	High
(15)	AlMahmoud et al. (2019)	Retrospective observational cohort	6/9	Moderate
(16)	Krishnaiah et al. (2006)	Population-based cross-sectional study	8/9	High
(17)	Mir et al. (2020)	Retrospective cross-sectional nationwide database study	8/9	High
(18)	Kwon et al. (2020)	Nationwide population-based cross-sectional study	8/9	High
(20)	Alik et al. (2023)	Retrospective cross-sectional national trauma database study	8/9	High
(21)	Onakpoya et al. (2010)	Multicenter retrospective clinical study	6/9	Moderate
(22)	Chocron et al. (2020)	Retrospective chart review	6/9	Moderate
(23)	Halawa et al. (2021)	Retrospective cohort study using national injury database	8/9	High
(24)	Cohen et al. (2019)	Retrospective chart review	5/9	Moderate
(27)	Emami-Naeini et al. (2013)	Retrospective case series	5/9	Moderate
(28)	Cillino et al. (2008)	Retrospective hospitalized cohort	6/9	Moderate
(29)	Cao et al. (2012)	Retrospective multicenter hospitalized cohort	7/9	High
(30)	Pandita et al. (2012)	Retrospective study	6/9	Moderate
(31)	Anant et al. (2025)	Multicenter retrospective cohort	8/9	High
(32)	Chen et al. (2024)	Retrospective hospital-based cohort	7/9	High
(33)	Yılmaz et al. (2025)	Hospital-based retrospective cross-sectional study	6/9	Moderate
(34)	Smith et al. (2005)	Retrospective occupational cohort/hospitalization analysis	8/9	High
(35)	Haring et al. (2016)	Nationwide emergency department database study	8/9	High
(36)	Hashemi et al. (2023)	Population-based cross-sectional study	8/9	High
(37)	Sahraravand et al. (2018)	Prospective hospital-based cohort	7/9	High
(38)	Uzel et al. (2024)	Retrospective tertiary-center cohort	6/9	Moderate
(39)	Lu et al. (2025)	Retrospective tertiary surgical cohort	7/9	High
(40)	Guzmán-Almagro et al. (2021)	Single-center retrospective cohort	6/9	Moderate
(41)	Siddiqui et al. (2021)	Retrospective national inpatient database cohort	8/9	High
(42)	Hoskin et al. (2021)	International multicenter registry cohort	8/9	High
(43)	Puodžiuvienė et al. (2021)	Retrospective clinical cohort	6/9	Moderate
(44)	Kim et al. (2025)	Registry-based multicenter retrospective observational study	8/9	High
(45)	Okamoto et al. (2019)	Multicenter retrospective case-series/cohort	7/9	High
(46)	Sii et al. (2018)	Prospective observational surveillance study	8/9	High
(47)	Celik et al. (2025)	Retrospective cohort	7/9	High
(48)	Borjan et al. (2025)	Retrospective cohort	6/9	Moderate
(49)	Rmili et al. (2024)	Retrospective pediatric cohort	7/9	High
(50)	Shah et al. (2020)	Prospective pediatric cohort	7/9	High
(51)	Ojabo et al. (2015)	Retrospective pediatric case study/cohort	5/9	Moderate
(52)	D’Antone et al. (2021)	Retrospective cross-sectional pediatric hospital study	6/9	Moderate
(53)	Abu-Ain et al. (2025)	Retrospective pediatric tertiary cohort	6/9	Moderate
(54)	Israeli et al. (2024)	Retrospective descriptive pediatric study	6/9	Moderate
(55)	Ashby et al. (2023)	Multicenter population-based pediatric cohort	8/9	High
(56)	Boret et al. (2020)	Retrospective pediatric cohort	7/9	High
(57)	Matsa et al. (2018)	Retrospective national emergency department cohort	8/9	High
(58)	Bućan et al. (2017)	Retrospective pediatric admission cohort	7/9	High
(59)	Patel et al. (2023)	Cross-sectional national emergency department database study	8/9	High
(60)	Ferguson et al. (2019)	Nationwide retrospective review	8/9	High
(61)	Sahu et al. (2024)	Prospective observational study	7/9	High
(62)	Shan et al. (2025)	GBD-based retrospective burden analysis	8/9	High

This table presents the methodological quality assessment of the observational studies formally included in the systematic review using the Newcastle–Ottawa Scale, adapted where appropriate for cross-sectional, registry-based, and database studies. Overall NOS scores and quality ratings are shown.

**Table 2:** Summary of primary human studies included in the systematic review.

**Ref**	**Study**	**Country/setting**	**Study design/population**	**Primary study focus**	**Principal sex-related finding relevant to ocular trauma**
(14)	Koo et al. (2005)	United States	Retrospective open-globe injury cohort	Etiology and outcomes of open-globe injuries	Men predominated; men had more penetrating/projectile injuries; women had more blunt globe rupture, often fall-related
(15)	AlMahmoud et al. (2019)	United Arab Emirates	Retrospective observational surgical trauma cohort	Epidemiology of eye injuries requiring hospitalization/surgery	Strong male predominance; male injuries were more often occupational; female injuries more often occurred at home
(16)	Krishnaiah et al. (2006)	India	Population-based study	Ocular trauma in a rural population	Demonstrated male predominance in ocular trauma at community level
(17)	Mir et al. (2020)	United States	National/database open-globe study	Characteristics of open-globe injuries	Open-globe injuries were more common in males; severe injury burden greater in men
(18)	Kwon et al. (2020)	South Korea	Nationwide population-based study	Incidence and seasonality of major ocular trauma	Male incidence was higher across major ocular trauma categories including open-globe injury and IOFB removal
(20)	Alik et al. (2023)	United States	Epidemiologic database study	Intimate partner violence-related ocular trauma	Women predominated in IPV-related ocular trauma; home setting was common
(21)	Onakpoya et al. (2010)	Nigeria	Observational cohorts of older adults	Ocular trauma among the older adults	Falls and home-related injuries were important in older adults; women relatively more represented in fall-related patterns
(22)	Chocron et al. (2020)	United States	Urban older adult cohort	Patterns of ocular trauma in older adult patients	Trauma in older adults showed more fall-related mechanisms, especially among women
(23)	Halawa et al. (2021)	United States	Population-based/administrative study	Fall-related eye injury among older adults	Older women were disproportionately affected by fall-related ocular trauma
(24)	Cohen et al. (2019)	Canada	Clinical observational study	IPV as mechanism of ocular injury in women	Highlights violence-related ocular trauma in women
(27)	Emami-Naeini et al. (2013)	United States	Retrospective urban open-globe cohort	Gender disparities in open-globe injuries	Men predominated overall; women were older and more likely to have fall-related ruptures
(28)	Cillino et al. (2008)	Italy/Mediterranean area	Retrospective hospitalized cohort	Epidemiology and visual outcomes of ocular trauma	Male predominance among hospitalized cases; sex differences in mechanism and outcome patterns
(29)	Cao et al. (2012)	China	Retrospective hospitalized cohort	Epidemiology of hospitalized ocular trauma	Strong male predominance; occupational and tool-related mechanisms common in men
(30)	Pandita and Merriman (2012)	New Zealand	Retrospective epidemiologic study	Ocular trauma epidemiology	Male predominance across trauma presentations
(31)	Anant et al. (2025)	United States	Multi-center study	Mechanisms of open-globe injury	Mechanism profiles differed by sex, with more high-energy patterns in men
(32)	Chen et al. (2024)	China	Retrospective clinical cohort	Epidemiologic and clinical characteristics of open-globe injuries	Male predominance; severe and posterior injuries common in men
(33)	Yılmaz (2025)	Türkiye	Public hospital cohort	Epidemiologic and clinical findings of ocular trauma	Male predominance and work-related patterns were prominent
(34)	Smith et al. (2005)	United States	Occupational hospitalization study	Whether occupation explains gender differences in eye injury hospitalization	Men had higher rates even after adjustment for occupational group, suggesting behavioral/protective factors beyond job category
(35)	Haring et al. (2016)	United States	Emergency department database study	Ocular injury in the United States	Male predominance persisted across ED presentations
(36)	Hashemi et al. (2023)	Iran	Population-based study of older adults	Epidemiology of ocular trauma in older adults	Fall-related injury is important in older adults; women are relatively more vulnerable in older age groups
(37)	Sahraravand et al. (2018)	Finland	Ocular trauma study in older adults	Ocular trauma in older adults in Finnish	Supports age-related shift toward fall-related mechanisms
(38)	Uzel et al. (2024)	Germany	Tertiary older adult cohort (>90 years)	Fall-related ocular trauma in very old patients	Highlights extreme old-age fall-related trauma, relevant to female vulnerability in older age
(39)	Lu et al. (2025)	China	Retrospective tertiary hospital cohort	Epidemiology and predictive modeling of visual outcomes	Strong male predominance; severe posterior segment complications associated with worse outcomes
(40)	Guzmán-Almagro et al. (2021)	Spain	Single-center retrospective open-globe cohort	Open-globe injury characteristics	Sex differences in anterior/posterior injury patterns and outcomes
(41)	Siddiqui et al. (2021)	United States	Inpatient epidemiology study	Inpatient open-globe injuries	Men predominated in hospitalized severe open-globe trauma
(42)	Hoskin et al. (2021)	International multicenter	Multicenter epidemiology/outcomes study	Epidemiology and outcomes of open-globe injuries	Supports severe open-globe burden and prognostic factors; men predominated
(43)	Puodžiuvienė et al. (2021)	Lithuania	Clinical cohort	Characteristics, outcomes, prognostic factors of OGI	Men predominated; posterior involvement worsened prognosis
(44)	Kim et al. (2025)	South Korea/multicenter registry	Registry-based city-wide study	Causative activities and prognostic factors of OGI	Activity/mechanism patterns differed; men were more represented in severe trauma
(45)	Okamoto et al. (2019)	Japan	Clinical cohort	Clinical characteristics and outcomes of OGI	Male predominance and severe injury burden in men
(46)	Sii et al. (2018)	United Kingdom	Pediatric trauma study	Demographics and mechanisms in pediatric ocular trauma	Boys predominated; mechanism patterns differed by age
(47)	Celik et al. (2025)	Türkiye	Regional open-globe cohort	Demographics and visual prognosis of OGI	Men predominated; severe open-globe injury burden high
(48)	Borjan et al. (2025)	Croatia	Cohort study	OGI characteristics and prognostic value of OTS	Men predominated; severe injury patterns linked to poorer outcomes
(49)	Rmili et al. (2024)	Tunisia	Retrospective pediatric tertiary-center study	Pediatric ocular trauma epidemiology and outcomes	Boys predominated in pediatric ocular trauma
(50)	Shah et al. (2020)	India	Prospective pediatric cohort	Closed-globe pediatric trauma epidemiology	Boys had higher injury burden; mechanism varied by age
(51)	Ojabo et al. (2015)	Nigeria	Pediatric open-globe cohort	Epidemiology, etiology, outcomes in children	Severe pediatric OGI disproportionately affected boys
(52)	D’Antone et al. (2021)	Colombia	Public hospital pediatric cohort	Childhood ocular trauma epidemiology	Boys predominated in childhood ocular trauma
(53)	Abu-Ain et al. (2025)	Jordan	Tertiary pediatric open-globe cohort	Epidemiology of pediatric OGI	Boys disproportionately affected
(54)	Israeli et al. (2024)	Israel	Tertiary hospital pediatric study	Characteristics of pediatric ocular trauma	Male predominance in pediatric trauma
(55)	Ashby et al. (2023)	Population-based cohort	Pediatric ocular trauma incidence and features	Population-based pediatric ocular trauma	Boys had higher incidence
(56)	Boret et al. (2020)	France	Pediatric cohort	Pediatric ocular trauma characteristics and outcomes	Boys predominated; mechanisms varied by context
(57)	Matsa et al. (2018)	United States	ED trends study	Pediatric acute ocular injury visits	Boys predominated in ED presentations
(58)	Bućan et al. (2017)	Croatia	Retrospective pediatric admission cohort	Ocular trauma requiring hospital admission	Severe admitted pediatric trauma disproportionately affected boys
(59)	Patel et al. (2023)	United States	Multi-center cross-sectional ED study	Sports-related ocular trauma	Majority of injuries occurred in males, especially adolescents
(60)	Ferguson et al. (2019)	New Zealand	National childhood injury study	Childhood ocular trauma	Boys showed higher injury rates and greater risk of visual impairment
(61)	Sahu et al. (2024)	India	Prospective observational emergency cohort	Pattern and clinical profile of ocular trauma	Demonstrates setting-specific mechanism patterns; useful for socioeconomic/contextual comparison
(62)	Shan et al. (2025)	Global burden study	Global/regional/national burden analysis	Gender disparity of intraocular foreign bodies	IOFB burden disproportionately affected males across regions

This table summarizes the main characteristics of the original human epidemiologic and clinical studies formally included in the systematic review, including author, year, country or setting, study design, population or sample, primary study focus, and the principal sex-related findings relevant to ocular trauma.

**Table 3:** Contextual, biologic, and mechanistic references cited for interpretation in the Introduction and Discussion.

Ref	Study	Category	Main contextual contribution
(1)	Li et al. (2023)	Global burden/epidemiologic context	Global incidence and disability burden of eye injury
(2)	Négrel and Thylefors (1998)	Global burden/epidemiologic context	Classic estimates of global impact of eye injuries
(3)	Gilbert and Foster (2001)	Public health/childhood blindness context	Broader burden of blindness in children and low-resource settings
(4)	Glynn et al. (1988)	Epidemiologic background	Community incidence of eye injuries in adults
(5)	Wong et al. (2000)	Epidemiologic background	Prevalence and incidence of ocular trauma in Beaver Dam Eye Study
(6)	McCarty et al. (1999)	Epidemiologic background	Ocular trauma epidemiology in Australia
(7)	Tielsch et al. (1989)	Hospitalization trend background	Time trends in hospitalized ocular trauma
(8)	Voon et al. (2001)	Tertiary hospital epidemiologic background	Emergency-service perspective on ocular trauma
(9)	Morris et al. (2014)	Incidence background	Serious eye injury incidence in Scotland
(10)	He et al. (2022)	General descriptive background	Characteristics of ocular trauma in the United States
(11)	Vought et al. (2025)	Narrative review/occupational context	Occupational eye injury prevention context
(12)	Thylefors (1992)	Classic epidemiologic context	Broad epidemiologic patterns of ocular trauma
(13)	Mir et al. (2024)	Systematic review/regional context	Context on factors affecting ocular trauma in Iran
(25)	Mansouri et al. (2007)	Contextual clinical report	Domestic ocular injuries; not formal sex-comparison dataset
(26)	Kuhn et al. (1996)	Classification/terminology	Standardized classification of ocular trauma
(63)	Tripathi et al. (2019)	Mechanistic/biologic context	Whether sex is a biologic variable in corneal wound healing
(64)	Sen et al. (2015)	Ocular inflammation/biologic context	Gender disparities in ocular inflammatory disorders
(65)	Nuzzi et al. (2018)	Hormonal/retinal context	Gonadal hormones and retinal disorders
(66)	Suzuki et al. (2001)	Receptor biology	Sex steroid hormone receptor expression in cornea
(67)	Wickham et al. (2000)	Receptor biology	Androgen, estrogen, and progesterone receptor mRNAs in the eye
(68)	Kumar et al. (2005)	Experimental neuroprotection	Estrogen analogue neuroprotection in retinal ganglion cells
(69)	Kaja et al. (2003)	Experimental retinal protection	Estrogen and retinal anti-apoptotic effects
(70)	Engeland et al. (2009)	Wound-healing biology	Sex hormones and mucosal wound healing
(71)	Klein and Flanagan (2016)	Immunologic sex differences	Sex differences in immune responses
(72)	Kumar et al. (2008)	Retinal neuroprotection context	Estrogens and neuroprotection in retinal diseases
(73)	Prokai-Tatrai et al. (2025)	Experimental retinal neuroprotection	Retina-targeted estradiol in optic nerve crush model
(74)	Prokai-Tatrai et al. (2013)	Experimental glaucoma model	Estradiol eye drops protecting retinal ganglion cell layer
(75)	Prokai-Tatrai et al. (2020)	Drug delivery/retinal hormone targeting	Retina-targeted estradiol delivery with DHED prodrug

This table summarizes the references cited to support the epidemiologic, biologic, and conceptual interpretation of sex-related differences in ocular trauma. These studies were used for contextual interpretation in the Introduction and Discussion and were not part of the formal systematic review dataset.

### Narrative synthesis of included studies

#### Gender differences in injury mechanisms

The included studies consistently demonstrate a marked male predominance in ocular trauma, particularly in injuries associated with occupational, recreational, and high-energy mechanisms (14–17, 27–35). Across hospital-based, population-based, and database studies, males were more frequently injured in workplace, industrial, and tool-related settings, whereas females were relatively more represented in domestic and fall-related injury contexts (14–16, 21, 27–29, 33, 36). This pattern suggests that sex differences in ocular trauma are closely linked to differences in exposure environment and mechanism of injury.

The relationship between male sex and occupational trauma was particularly evident in studies of severe and surgically treated injuries. In a retrospective analysis from the United Arab Emirates, AlMahmoud et al. (15) found that 89% of injured patients were male and that more than half of all injuries occurred in workplace environments; male injuries were more likely to be work-related, whereas female injuries were more often sustained at home. Similar patterns were observed in other cohort studies, where males were disproportionately represented among injuries involving sharp objects, projectiles, and occupational activities (14, 27, 29–33). These findings support the interpretation that occupational exposure is a major driver of male-predominant ocular trauma, particularly in high-energy and penetrating injuries.

However, occupational exposure alone does not fully explain the observed sex disparity. Smith et al. (34) showed that sex differences in work-related eye injury hospitalization persisted even after adjustment for occupational group, suggesting that behavioral factors, such as risk-taking tendencies and inconsistent protective eyewear use, may also contribute. This interpretation is further supported by national and population-based studies showing continued male predominance across broader injury settings beyond the workplace alone (16, 18, 35).

In contrast, females were more commonly represented in domestic, fall-related, and interpersonal violence-related injury patterns. Koo et al. (14) and Emami-Naeini et al. (27) both found that women with open-globe injuries were more likely to sustain globe rupture after falls, whereas men were more likely to sustain penetrating or projectile trauma associated with work or recreation. Similarly, older adult cohorts showed a greater relative contribution of falls and home-based injury among women, indicating that the mechanism profile shifts with age and differs by sex (21–23, 36–38). Interpersonal violence is another important exception to the usual male predominance in ocular trauma, as Alik et al. (20) reported that ocular injuries related to intimate partner violence occurred predominantly in females. Taken together, these findings indicate that sex differences in ocular trauma are not only quantitative, but also qualitative, with distinct mechanism profiles in males and females.

### Type and severity of injury

Marked differences exist between males and females in both the type and severity of ocular trauma, largely reflecting variation in exposure, injury mechanism, and risk behavior (14, 15, 17, 18, 27, 31–33, 39–48). Population-based data consistently demonstrate a substantially higher incidence of major ocular trauma categories among males. In a nationwide Korean study, Kwon et al. (18) reported higher male incidence across hyphema, orbital blowout fracture, open-globe injury, and intraocular foreign body removal, highlighting a greater burden of both blunt and penetrating trauma among men at the population level.

Clinical cohort studies further support sex-related differences in injury mechanism and severity. In a retrospective study of open-globe injuries, Koo et al. (14) found that men were significantly more likely to sustain penetrating injuries, often associated with projectile trauma, whereas women were more likely to sustain globe rupture related to blunt trauma and falls. Similar findings were reported by Emami-Naeini et al. (27), who also observed that males predominated in open-globe injury overall, while females were more frequently represented in fall-related rupture patterns. These findings suggest that occupational and recreational exposures contribute to more severe penetrating injuries among males, whereas fall-related blunt trauma represents a more common severe injury pattern among females.

Hospital-based and registry studies further reinforce the predominance of severe ocular injuries among males. In a retrospective analysis of surgically treated trauma, AlMahmoud et al. (15) reported that 89% of injured patients were male and that many injuries were work-related. Lu et al. (39) similarly found that 80.8% of surgically treated ocular trauma patients were male. Large registry and database studies of open-globe injury also showed strong male predominance and emphasized the contribution of high-energy, penetrating, and work-associated injury mechanisms in men (17, 31–33, 41–45, 47, 48). Collectively, these studies indicate that males are more likely to experience severe penetrating injuries and occupational trauma, whereas females more commonly sustain blunt trauma related to falls or domestic accidents.

### Anatomical patterns of ocular injury

Sex-related differences in injury mechanisms are reflected in distinct anatomical patterns of ocular involvement. Male patients more frequently sustain injuries involving deeper ocular structures and the posterior segment, including vitreous hemorrhage, retinal detachment, and other complications associated with irreversible visual loss (17, 32, 39, 40, 42, 43, 47, 48). In addition, intraocular foreign bodies are more common among males in population-based and open-globe cohorts, further increasing the risk of complex surgical management and poorer visual outcomes (17, 18, 41, 44).

In a large retrospective cohort of surgically treated ocular trauma, Lu et al. (39) reported high rates of severe posterior segment complications, including vitreous hemorrhage, retinal detachment, and choroidal detachment, all of which were significantly associated with worse visual prognosis. Similarly, registry-based and multicenter open-globe injury studies support the view that high-energy trauma in males is frequently associated with more severe posterior involvement and more complex injury patterns (17, 31–33, 42, 44, 47, 48).

In contrast, ocular trauma in females more frequently involves anterior segment structures, including the cornea, anterior chamber, and crystalline lens (14, 40). These injuries are often associated with lower-energy blunt trauma, such as falls or domestic accidents, and may present as corneal lacerations, hyphema, lens dislocation, or traumatic cataract. Compared with posterior segment trauma, anterior segment injuries are often more amenable to timely medical or surgical management, although severe blunt trauma can still result in globe rupture or posterior extension, particularly in older adult patients (14, 38, 40, 45).

Further evidence of sex-related anatomical differences was demonstrated in the open-globe cohort reported by Koo et al. (14), in which zone 1 corneal injuries were more common in men, whereas more posteriorly extending injuries were more common in women. These findings suggest that penetrating injuries in males often involve the anterior cornea, while blunt trauma in females may result in rupture patterns with greater posterior extension, contributing to differences in injury phenotype and clinical outcome between sexes.

### Pediatric considerations

Sex differences in ocular trauma are evident even in childhood, with consistently higher injury rates among boys than girls across a wide range of study designs and settings (46, 49–60). This disparity is likely related to behavioral and environmental factors, including greater participation in sports, outdoor activity, projectile play, and unsupervised risk exposure among boys.

Supporting this pattern, a large U.S. emergency department-based study of sports-related ocular trauma reported that 79% of injuries occurred in males, with the highest incidence observed among adolescents aged 12–17 years, underscoring the role of recreational exposure in driving male-predominant injury patterns (59). Population-level data from New Zealand similarly demonstrated a male predominance in pediatric ocular trauma, with boys showing higher injury rates and greater risk of permanent visual impairment than girls (60). Hospital-based pediatric cohorts also consistently found that severe ocular trauma requiring admission or operative management disproportionately affected boys (46, 49, 51, 53, 56, 58).

At the same time, the mechanism profile in pediatric trauma appears to vary by age and setting. Sports and recreation account for a substantial proportion of injuries in older boys, whereas home-based trauma, object-related injury, and accidental play appear more prominent in younger children (46, 50–53, 56–59). These findings suggest that sex differences in pediatric ocular trauma are both real and context-dependent, and that prevention strategies should be age-targeted as well as sex-informed.

## Discussion

This systematic review with narrative synthesis demonstrates consistent and clinically meaningful sex-based differences in ocular trauma across epidemiology, injury mechanisms, severity, anatomical patterns, and outcomes. The most reproducible finding across the included literature is the predominance of males in most ocular trauma settings (11–13). For example, Thylefors et al. (12) reported that up to 84% of ocular injuries occur in men, with a male-to-female ratio of approximately 4:1 in pediatric populations, while Lu et al. (39) reported a male predominance of 80.8% in a large cohort. However, the magnitude of this difference varies across settings and injury types, suggesting that differences in exposure patterns likely play a major role. Importantly, this pattern was not uniform across all study designs, indicating that sex differences in ocular trauma are shaped not only by biologic factors, but also by the context in which injuries are captured and reported.

The included evidence consistently demonstrates sex-related differences in injury mechanisms and environments. Males are more frequently injured in occupational, industrial, sports-related, and recreational settings, whereas females are relatively more represented in domestic, fall-related, blunt, and interpersonal violence–related injuries (14, 15, 20–23, 27, 28, 36, 61). For instance, Alik et al. (20) reported that females accounted for 62.3% of intimate partner violence–related ocular trauma, with 74.4% of injuries occurring at home and higher odds of home-related injury (OR 1.91, 95% CI 1.60–2.29). Similarly, AlMahmoud et al. (15) found that 57.3% of male injuries occurred in occupational settings, compared to 88.2% of female injuries occurring at home. Importantly, occupational exposure alone does not fully explain these disparities. Smith et al. (34) demonstrated that work-related eye injury rates remained significantly higher in men compared to women (29.9 vs. 9.3 per 100,000 person-years; IRR 3.2, 95% CI 2.7–3.8), even after adjustment for occupational group (adjusted IRR ≈ 2.2). Nationwide population-based data further support this pattern, with higher male incidence across major injury categories, including hyphema (29.28 vs. 7.60), orbital blowout fractures (18.72 vs. 4.45), open-globe injuries (3.15 vs. 0.83), and intraocular foreign body removal (0.68 vs. 0.09) (18). Taken together, these studies suggest that the observed male predominance reflects a combination of differential exposure, risk behavior, and protective practices rather than occupational assignment alone. At the same time, the greater representation of females in domestic and fall-related trauma highlights that sex differences in ocular injury are qualitative as well as quantitative, with distinct mechanism profiles in each group.

Sex-based differences also extend to injury type and severity. Males are more likely to sustain high-energy penetrating injuries, intraocular foreign bodies, and posterior segment involvement, whereas females more commonly experience blunt trauma. Koo et al. (14) reported that males accounted for 78.6% of open-globe injuries and were more likely to sustain penetrating injuries (69.9%) related to projectile mechanisms (54.9%), whereas females more commonly experienced blunt globe rupture (68.1%), often associated with falls. These mechanism-driven differences likely explain the disproportionate representation of males among severe and surgically treated trauma cases (14, 39, 40). This interpretation also helps explain why studies from tertiary referral centers and surgical cohorts often report a stronger male predominance than community-based or emergency department-based studies, as high-energy penetrating injuries are more likely to require specialized care.

Outcome data, however, are more nuanced. While some studies associate male sex with poorer visual outcomes due to a higher prevalence of high-energy penetrating injuries, others demonstrate that females—particularly older women—may present with worse initial visual acuity related to fall-associated trauma but achieve comparable final outcomes (14, 27). These findings suggest that prognosis is driven more by injury mechanism and anatomical involvement than by sex alone. In other words, sex appears to function partly as a marker of differing exposure and injury phenotype, rather than as an independent determinant of outcome in all settings.

Geographic and socioeconomic context further influences ocular trauma patterns. Studies from developing regions report a predominance of road traffic–related injuries (67.7%), with most cases involving closed-globe trauma (87.7%) and relatively preserved visual acuity (69.4%) (61). In contrast, high-income settings more frequently report occupational and high-energy trauma. Global data further demonstrate that severe injuries such as intraocular foreign bodies disproportionately affect males, with higher incidence rates (500.35 vs. 206.48 per 100,000), particularly among working-age individuals, and with overall case numbers increasing by 33%, especially in low-SDI regions (up to 129%) (14, 62). These findings highlight the influence of socioeconomic conditions, infrastructure, and access to safety measures in shaping injury risk. They also suggest that between-study differences are not simply methodological noise, but may reflect real variation in occupational structure, transportation safety, supervision, and access to protective equipment across settings.

Study design represents an important source of heterogeneity. Population-based studies provide robust estimates of incidence and sex distribution but often lack detailed clinical information, whereas hospital-based and open-globe cohorts offer detailed insights into mechanism, anatomy, and outcomes but may overrepresent severe injury. This distinction explains why male predominance is more pronounced in severe and surgically managed cohorts, while appearing less marked in community-based studies (14, 15, 27, 28, 39, 40, 63). This difference in case capture is critical when interpreting the literature, because it means that apparent inconsistencies across studies may arise from referral patterns and injury severity thresholds rather than true disagreement in sex-related risk.

Age further modifies these patterns. In pediatric populations, boys account for approximately 65–75% of ocular trauma cases and are more frequently injured during sports and recreational activities (58–60). In contrast, older adult populations demonstrate a shift toward low-energy mechanisms, particularly falls, which account for up to 50–60% of injuries and disproportionately affect women (21–23, 36). These findings support a clear age–sex interaction, with male predominance strongest in younger and working-age populations and female vulnerability increasing in older age groups. This age-related shift likely reflects changing exposure profiles across the life course, with recreational and occupational risk dominating earlier years and fall-related vulnerability becoming more important in later life.

Beyond environmental and behavioral factors, biologic sex may also influence injury response. Experimental evidence suggests that estrogen exerts anti-inflammatory and neuroprotective effects, whereas testosterone has been associated with enhanced inflammatory responses and delayed healing (64–75). These findings are biologically plausible and hypothesis-generating, but their direct clinical relevance to ocular trauma outcomes in humans remains uncertain. The presence of sex hormone receptors in ocular tissues further supports biological plausibility; however, current evidence remains largely preclinical, and its clinical relevance to ocular trauma outcomes requires further investigation. Accordingly, biologic mechanisms should currently be viewed as a possible contributing layer of explanation rather than a definitive explanation for the epidemiologic patterns observed in clinical studies.

These findings have important implications for prevention and clinical care. Sex-informed preventive strategies, including promotion of occupational safety and protective eyewear use among males, particularly given that up to 90% of serious eye injuries are preventable, as well as fall prevention and domestic safety interventions among females, may significantly reduce injury burden. Public health efforts targeting education, awareness, and access to protective equipment are especially critical in lower-resource settings. Despite clear epidemiologic differences, current management remains largely sex-neutral, highlighting an opportunity for improved risk stratification and the development of more personalized approaches to ocular trauma care.

### Limitations

This review has several limitations. First, the included studies were heterogeneous in design, populations, clinical settings, injury classifications, and reported outcomes, which limited direct comparability and precluded meta-analysis. Second, sex-disaggregated reporting was inconsistent across studies, and many articles did not provide sufficiently detailed data on age, mechanism, severity, management, or outcome to allow uniform subgroup analysis. Third, much of the available evidence was derived from retrospective, hospital-based, or surgically treated cohorts, which may overrepresent more severe trauma and may not fully reflect the epidemiology of ocular injury at the community level. Fourth, the formal search was restricted to PubMed/MEDLINE and manual reference screening, and relevant studies indexed exclusively in other databases may therefore have been missed. Fifth, the evidence concerning biologic sex-related mechanisms remains largely experimental or preclinical, limiting firm conclusions about their clinical relevance in human ocular trauma. Finally, publication bias, selective outcome reporting, and variable methodological quality across the included studies cannot be excluded.

## Conclusion

Ocular trauma demonstrates significant sex-based differences across epidemiology, mechanisms, injury patterns, and outcomes. Males are more likely to sustain high-energy, severe injuries related to occupational and behavioral factors, whereas females more commonly experience domestic and fall-related trauma. These differences are further influenced by age, geographic, and socioeconomic factors. Recognition of these patterns is essential to guide targeted prevention strategies and improve patient outcomes. Future research should focus on integrating sex, age, and socioeconomic variables to develop more personalized and effective approaches to ocular trauma prevention and management.
